# Inflammation in Older Poles with Localized and Widespread Chronic Pain—Results from a Population-Based PolSenior Study

**DOI:** 10.3390/jcm13195870

**Published:** 2024-10-01

**Authors:** Anna Chudek, Przemysław Kotyla, Elżbieta Kozak-Szkopek, Małgorzata Mossakowska, Katarzyna Wieczorowska-Tobis, Joanna Sulicka-Grodzicka, Magdalena Olszanecka-Glinianowicz, Jerzy Chudek, Aleksander J. Owczarek

**Affiliations:** 1 Health Promotion and Obesity Management Unit, Department of Pathophysiology, Faculty of Medical Sciences in Katowice, Medical University of Silesia in Katowice, 40-752 Katowice, Poland; magolsza@gmail.com (M.O.-G.); aowczarek@sum.edu.pl (A.J.O.); 2 Department of Internal Medicine, Rheumatology and Clinical Immunology, Faculty of Medical Sciences in Katowice, Medical University of Silesia in Katowice, 40-635 Katowice, Poland; 3 Department of Geriatric Nursing, Medical University of Warsaw, 02-091 Warsaw, Poland; elzbieta.kozak-szkopek@wum.edu; 4 Department of Internal Diseases and Cardiology, Centre for Management of Venous Thromboembolic Disease, Medical University of Warsaw, 02-005 Warsaw, Poland; 5 Study on Ageing and Longevity, International Institute of Molecular and Cell Biology, 02-109 Warsaw, Poland; mmossakowska@iimcb.gov.pl; 6 Geriatric Unit, Department of Palliative Medicine, University of Medical Sciences, 61-701 Poznan, Poland; kwt@tobis.pl; 7 Department of Rheumatology and Immunology, Jagiellonian University Medical College, 30-698 Cracow, Poland; joanna.sulicka-grodzicka@uj.edu.pl; 8 School of Infection & Immunity, College of Medical, Veterinary and Life Sciences, University of Glasgow, Glasgow G12 8QQ, UK; 9 Department of Internal Medicine and Oncological Chemotherapy, Medical University of Silesia, 40-029 Katowice, Poland; chj@poczta.fm

**Keywords:** chronic pain, chronic widespread pain, chronic regional pain, inflammation, older adults, epidemiology

## Abstract

**Background**: Inflammation leads to a decrease in the excitation threshold and the sensitization of peripheral nociceptors. However, little is known about the effect of inflammation on the sensing of regional (CRegP) and widespread chronic pain (CWP) in older adults. This analysis aimed to characterize the prevalence and associates of both types of chronic pain in a population-based cohort. **Methods**: Our analysis was based on the Polish nationwide PolSenior study database. We excluded participants with moderate-to-severe dementia. Respondents answered questions concerning the occurrence of pain in 10 regions. CWP was defined as chronic pain present in the axial region (neck, upper back, lower back) and any part of both the lower (lower leg, hip, knee, foot) and upper (shoulder, hand) extremities. Inflammatory status was divided into three subgroups: no inflammation (CRP < 3 mg/dL), mild inflammation (CPR 3–10 mg/dL and IL-6 < 10 ng/mL), and significant inflammation (CRP ≥ 10 mg/dL or IL-6 ≥ 10 ng/mL). **Results**: CRegP was more frequent (33.9%) than CWP (8.8%). The occurrence of CWP was more frequent in subgroups with significant inflammation than in both subgroups with mild or no inflammation (11.4% vs. both 8.4%). Women (OR 3.67; 95% CI: 2.58–5.21) and subjects with major depression symptoms were more likely to experience CWP (OR 2.85; 95% CI: 1.68–4.82), while, malnourished participants were more likely to report CRegP (OR 2.00; 95% CI: 1.52–2.62). **Conclusions**: Significant inflammation is associated with increased occurrence of CWP in older adults. Female sex and major depression are the most significant associates of CWP, while malnutrition is the most significant associate of CRegP.

## 1. Introduction

Chronic pain occurs in 35–50% of community-dwelling older adults, including Poland [1,2], and impairs daily activity and mobility [3], as well as health-related quality of life [4]. Moreover, it increases the risk of falls and all their additional consequences [3,5]. In addition, the occurrence of chronic pain increases the utilization of health care services and the use and overuse of analgesics [6,7]. Inflammation related to degenerative joint changes and inflammation associated with other coexisting diseases decreases the excitation threshold and increases the sensitization of peripheral nociceptors [8,9,10]. Pain perception in osteoarthritis seems to be determined not only by local joint injuries, but also by neuroplastic changes in the nervous system, psychological and genetic factors, and metabolic diseases [11]. In addition, the loss of descending antinociceptive pathways is considered a central component of pain-sensing mechanisms [12].

Chronic pain is also associated with cardiovascular morbidity, in patients with and without osteoarthritis [13]. In a large cohort of 40–69-year-old participants of the UK biobank, chronic widespread pain (CWP), regardless of underlying pathology, was found to increase the risk of composite cardiovascular outcome (myocardial infarction, stroke, heart failure, or cardiovascular death) by 71% after an adjustment for age, sex, and cardiovascular risk factors [14].

Some individuals experience pain all over the body—so-called CWP [14]. Contrary to chronic localized pain, CWP refers to pain being on the left and right sides of the body, above and below the waist, and on the axial skeleton [15]. Its worldwide prevalence is estimated at 10.6% to 11.8% of adults, with higher occurrence in older people and women [16]. Furthermore, CWP is a common clinical condition and an early indicator of fibromyalgia syndrome (FMS) [17,18]. It is also frequent in subjects with rheumatoid arthritis and is associated with disease activity and considered as an indicator of inflammation severity [19]. According to the Framingham Heart Study, CWP increases the risk of cardiovascular death and should be a part of cardiovascular risk assessment [20].

The assessment of chronic pain was a part of the large national-based project on aging in Poland. The published analysis revealed a prevalence of chronic pain in 42.0% of respondents aged 65 years and over, and for it to be more common in women than men (48.6% vs. 35.8%) [2].

Contrary to the remarkable amount of epidemiological data concerning the prevalence of chronic regional pain (CRegP) in the very old population, there is a lack of studies analyzing the occurrence of CWP and its relation to inflammation in this specific population.

In the current study, we analyze the occurrence and associates of both chronic regional and widespread pain in relation to the intensity of inflammation.

## 2. Materials and Methods

### 2.1. Study Population

The analysis of chronic pain in community-dwelling older men and women was a part of the large nationwide, multicenter, interdisciplinary project on aging in Poland (PolSenior), performed between 2008 and 2010. Details of the study protocol were published elsewhere [21]. In brief, the project recruited 4979 subjects in six age cohorts (65–69 years, 70–74 years, 75–79 years, 80–84 years, 85–89 years, and 90 years or over) representative of the older Polish population. The protocol was approved by the Bioethics Committee of the Medical University of Silesia in Katowice (KNW-6501-38/I/08) and each subject or their caregiver signed informed consent.

Respondents answered the following questions concerning the occurrence of pain: Have you felt a nagging pain for longer than three months? Where it is localized (head, neck, shoulder, hand, upper back, lower back, lower leg, hip, knee, foot, other location)? Respondents had the possibility of identifying multiple sites of pain. Which of these places is the most painful? It was necessary to identify one of these places.

The pain severity, in the currently most painful place, was scored on the basis of the Visual Analog Scale (VAS) from 0 to 10, where 0 is no pain and 10 is the greatest pain imaginable.

**Figure 1:**
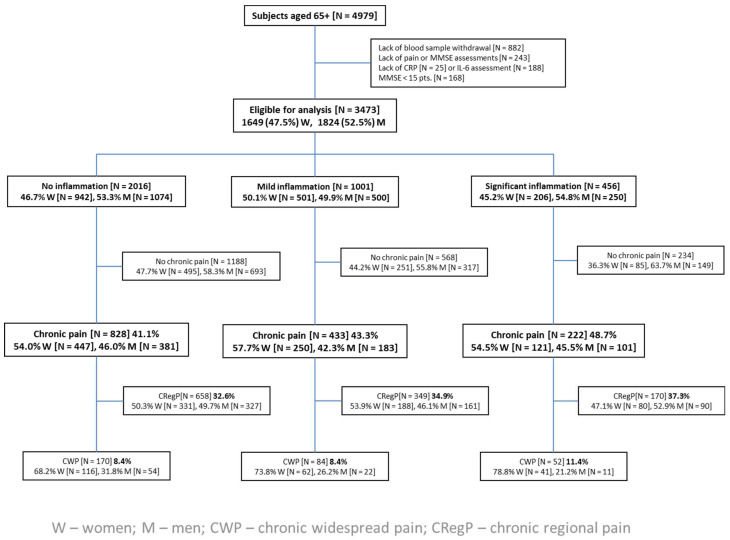
Flow chart of the analysis.

We excluded from the analysis participants suspected of moderate and severe dementia (Mini-Mental State Examination—MMSE <15 pts), those who refused blood sample donation, and those without measured serum levels of C-reactive protein (CRP) and interleukin 6 (IL-6), as seen in Figure 1. We analyzed the use of selected groups of medications: non-steroidal anti-inflammatory drugs—NSAIDs, aspirin, glucocorticoids—GCs, disease-modifying anti-rheumatic drugs—DMARDs, and xanthine oxidase inhibitors—XOIs, based on the participants’ list of medications.

### 2.2. Laboratory Assessments

All biochemical assessments (except total blood count) were performed in frozen samples in a central study laboratory in Warsaw and collaborating dedicated laboratories. Plasma interleukin-6 (IL-6) was measured by ELISA (R&D Systems, Minneapolis, MN, USA) with a limit of quantification (LoQ) of 0.04 pg/mL and a mean intra-assay and inter-assay coefficient of variance <7.8% and <7.2%, respectively. Serum CRP, creatinine, albumin, and uric acid were assessed by an automated system (Modular PPE, Roche Diagnostics GmbH, Mannheim, Germany) in a single laboratory with inter-assay coefficients of variability below 5.7%, 2.3%, 1.7%, and 1.7%, respectively. Anti-CCP antibodies were tested using the Anti-CCP Immunoscan RA ELISA kit (Euro Diagnostica AB, Malmö, Sweden). The serum titers ≥25 U/mL were scored as positive, as described previously [22].

### 2.3. Data Analysis

Chronic pain was defined as pain that lasted more than 3 months [2]. We distinguished two types of chronic pain similar to the definition from 2016: it is CWP if the pain was present in the axial region (neck, upper back, lower back) and any part of both lower (lower leg, hip, knee, foot) and upper (shoulder, hand) extremities; and it is CRegP when the criteria for CWP were not met [23]. The definition of CWP from 2016 was adopted as we did not assess left and right regions separately.

Concerning plasma concentrations of inflammatory markers, we distinguished participants with (1) no inflammation—if CRP levels were up to 3 mg/dL, (2) mild inflammation—if CPR levels were over 3 mg/dL but less than 10 mg/dL and IL-6 was below 10 ng/mL, and (3) significant inflammation—if both CRP and IL-6 levels were at least 10 mg/dL. The CRP cut-off of 10 mg/dL was based on international recommendations [24], while the cut-off value for IL-6 (10 ng/mL) was established as a value for the 95 percentiles of our study cohort.

The albumin level below the lower limit of the normal range (35 g/L) was scored as hypoalbuminemia. Hyperuricemia was defined as serum uric acid level above 6 mg/dL in women and 6.8 mg/dL in men (population sex-specific ranges corrected in men for the uric acid solubility in water), or the use of allopurinol—the only used XOI in the PolSenior cohort [25].

We use MMSE as a screening tool for dementia [26]. Participants with MMSE less than 15 pts were excluded from the analysis due to the low credibility of answers to the questions concerning the occurrence of pain [27].

A 15-item version of the Geriatric Depression Scale (GDS) was used as a screening tool for the assessment of depressive symptoms [28]. We distinguished subjects with major depression symptoms (>11 pts) among those subjects considered as having depression (>5 pts).

Functional status was analyzed using the Lawton Instrumental Activities of Daily Living Scale (IADL). The scores of 8–18 points were classified as a dependency, 19–23 points as a partial dependency, and 24 points as independence [29].

Nutritional status was defined according to WHO criteria, based on body mass index (BMI) as underweight (<18.5 kg/m^2^), normal weight (18.5–24.9 kg/m^2^), overweight (25.0–29.9 kg/m^2^), and obesity (≥30.0 kg/m^2^) [30]. The risk of malnutrition was assessed with the Mini Nutritional Assessment-Short Form (MNA-SF) and classified as: malnutrition (≤7 pts), risk of malnutrition (8–11 pts), and normal nutritional status (≥12 pts) [31].

Hypertension was diagnosed based on the average from four measurements per-formed on two separate visits or the use of antihypertensive medications, according to the 2013 ESH/ESC Guidelines for the Management of Arterial Hypertension [32], as described previously [33].

Diabetes was defined as a fasting plasma glucose level ≥126 mg/dL or the use of antidiabetic medications [34]. Congestive heart failure (CHF), coronary artery disease (CAD), chronic obstructive pulmonary disease (COPD), and asthma were scored based on the participants’ reports and hospital discharge cards.

Two diagnostic criteria of chronic kidney disease (CKD) were included into the analysis—decreased estimated glomerular filtration rate (GFR) and albumin-to-creatinine ratio (ACR) ≥30 mg/g. Values of eGFR were calculated based on the MDRD (Modification of Diet in Renal Disease) equation as appropriate for the used method of creatinine assessment. Age-adjusted eGFR cut-off was used—below 45 mL/min/1.73 m^2^—corresponding to stage G3b–G5 of CKD, according to NKF KDOQI guidelines [35].

### 2.4. Sociodemographic Variables

Data regarding gender, age, place of residence (rural vs. city dwellers), occupation in the past (white- or blue-collar workers, or farmers), income, occupational physical activity in the past, and smoking (active or former) were assessed based on the questionnaire. Personal income was divided into three categories: low (<100% of average), moderate (100–200% of average), and high (>200% of average), according to the average national retirement pension of 1000 PLN in 2009.

### 2.5. Statistical Analysis

Statistical analysis was performed using STATISTICA 13.0 PL (TIBCO Software Inc., Palo Alto, CA, USA) and R software v. 4.4.0 [R Core Team (2013). R: A language and environment for statistical computing. R Foundation for Statistical Computing, Vienna, Austria. URL: https://cran.r-project.org/ (accessed on 20 August 2024). Statistical significance was set at a *p*-value below 0.05. All tests were two-tailed. Imputations were not conducted for missing data. Nominal and ordinal data were expressed as percentages. Interval data were expressed as mean value ± standard deviation in the case of normal distribution. In the case of data with skewed or non-normal distribution, they were expressed as median, with lower and upper quartiles. The distribution of variables was evaluated by the Anderson–Darling test and the quantile–quantile (Q–Q) plot. The homogeneity of variances was assessed by the Levene test. Comparisons between the two groups were performed with either analysis of variances (ANOVA) with contrast post-hoc tests or with the log-linear analysis and χ^2^ tests. Risk factors of chronic pain occurrence as well as dependence, disability, and utilization of medical services were evaluated with univariable logistic regressions. Then, the best models of multivariable logistic regressions were calculated. Results were presented as corresponding odds ratios (OR) with confidence intervals (±95% CI) and *p*-values.

## 3. Results

### 3.1. Characteristics of the Sub-Study Population

There were 3473 subjects eligible for this analysis including 456 (13.1%) with significant inflammation, 1001 (28.8%) with mild inflammation, and 2016 (58.1%) without inflammation. Chronic pain was reported in 1483 subjects (42.7% of all studied); including 306 (8.8%) with CWP and 1177 (33.9% of all analyzed) with CRegP.

Chronic pain was more frequently reported by participants with significant inflammation than without (48.7% vs. 41.1%, *p* < 0.01). The difference in the occurrence of chronic pain between subjects with mild and significant inflammation was of borderline significance (*p* = 0.05). The occurrence of CWP in participants with significant inflammation (11.4%) was higher than with mild and no inflammation (both 8.4%), participants with significant inflammation were more likely to experience CWP–OR 3.67 (95% CI: 2.58–5.21, *p* < 0.001). In all subgroups with chronic pain, the most frequently reported pain was CRegP, with no difference between the subgroups despite the severity of inflammation (Figure 1).

### 3.2. Associates of Significant Inflammation

The occurrence of significant inflammation was associated with greater percentages of the oldest old, those at risk of malnutrition and malnourished subjects, active smokers, dependent according to the IADL, with professional physical activity (blue-collar, occupational physical activity in the past), and a history of hospitalization in the past 5 years. No inflammation was more frequent in rural dwellers, white-collar workers, subjects with high personal income, those physically active (reporting vigorous exercise in the past year), and beneficiaries of medical rehabilitation in the past 5 years (Table 1).

Among diseases more frequently co-occurring with significant inflammation were CHF, COPD, and depression. In addition, hypertriglyceridemia, hyperuricemia, increased ACR, hematuria, hypoalbuminemia, and anti-CCP seropositivity were more frequent in the subgroup with more severe inflammation. In addition, those subjects were more often prescribed with NSAIDs and GCs. Of note the utilization rates of GCs and DMARDs were low (Table 2).

### 3.3. Associates of Chronic Pain Related to Inflammation

A higher prevalence of chronic pain with accompanying significant inflammation was associated with active smoking, risk of malnutrition and being malnourished, dependency according to the IADL, occupational physical activity in the past, being blue- collar, having a low personal income, possessing a certificate of disability, having performed laboratory tests in the last 3 years, and being hospitalized in the past 5 years. Factors that may protect from chronic pain were high personal incomes, vigorous exercise in the past year, utilization of rehabilitation in the past 5 years, and management by specialists in the past 5 years (Table 1).

The chronic pain along with accompanying significant inflammation occurred more often in subjects with chronic pulmonary disease, depression, hypoalbuminemia, and low eGFR values. Additionally, in this subgroup, more frequent use of NSAIDs and GCs was found (Table 2).

### 3.4. Factors Related to the Occurrence of Chronic Pain

Univariate logistic regression analysis revealed several factors associated with the occurrence of chronic pain. The strongest ones included (from the most significant): malnutrition, major depression, hypoalbuminemia, female sex, chronic pulmonary disease, coronary artery disease, obesity, severity of inflammatory status, occupational physical activity in the past, and eGFR < 45 mL/min/1.73 m^2^. The protective effect was demonstrated for high personal income and vigorous exercise in the past year, as seen in Table 3.

The comparative analysis of factors related to CWP and CRegP revealed some differences. Major depression, female gender, and chronic pulmonary disease were much stronger risk factors for the occurrence of CWP than CRegP. Of note, diabetes was associated only with CWP, while hypoalbuminemia and low eGFR were only associated with CRegP as seen in Table 4. In multivariable analysis, female gender, major depression, malnutrition, coronary artery disease, chronic pulmonary disease, obesity, and significant inflammatory status were independent associates of the occurrence of chronic pain. Female gender, major depression, and chronic pulmonary disease were more associated with the occurrence of CWP. In contrast, both malnutrition and obesity were predictors for CRegP. Significant inflammation was an explanatory factor for CRegP. This association was not proved for CWP.

### 3.5. Dependence, Disability, and Utilization of Medical Services

**Figure 2:**
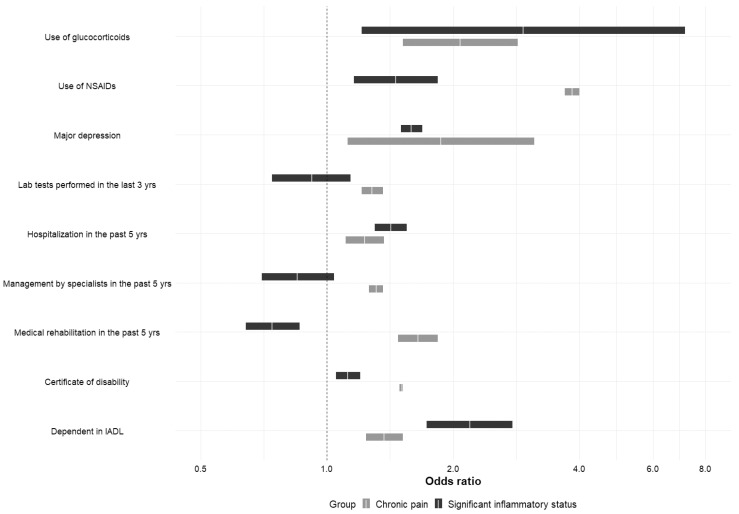
Dependence, disability, and utilization of medical services in relation to chronic pain and significant inflammatory status. Results of multivariable analyses adjusted for sex. DMARDS were not included in the analysis. Data are presented as OR with a 95% confidence interval. Abbreviations: IADL—The Lawton Instrumental Activities of Daily Living Scale; NSAIDS—non-steroidal anti-inflammatory drugs.

The comparative analyses of associates of chronic pain and significant inflammation, adjusted to sex, showed that subjects with chronic pain more frequently utilized NSAIDs, suffered from major depression symptoms, and benefited from medical rehabilitation, specialist care, and laboratory tests, as well as possessing a certificate of disability. Contrary to this, subjects with significant inflammation were more often prescribed with GCs, were more likely to have been hospitalized during the last 5 years, and were dependent according to IADL, as seen in Figure 2 (ORs presented in Table S1).

## 4. Discussion

In our subanalysis of the PolSenior study, we adopted the classification of chronic pain into CRegP and CWP, and searched for associates of both types of chronic pain in relation to inflammatory status. As a result of the performed analysis, we showed that CRegP was a much more frequent type of chronic pain in older adults. In addition, we showed that significant inflammation was more related to the occurrence of CWP and that being the female gender and having major depression were prominent associates of CWP. Contrary, the strongest correlate of CRegP was malnutrition.

Notably, CRegP was nearly four times more often than CWP (33.9% vs. 8.8%, respectively). To the best of our knowledge, studies comparing the prevalence of these types of chronic pain in older populations are missing. However, there are some epidemiological data concerning the prevalence of fibromyalgia, which is characterized by the occurrence of CWP between 2 and 4% of older adults [36], yet accurate data concerning the oldest population remains unknown [37]. According to Wolfe et al. [38], the prevalence of fibromyalgia was estimated at over 7% of women aged 60–79 living in the USA. A greater frequency of CWP in our study than reported for fibromyalgia is expected as not all of the causes of CWP met the criteria of this entity.

Our subanalysis of the PolSenior study analysis showed a very similar frequency of chronic pain being reported (42.7%) in older adults. The data are very similar to those as obtained in the whole population-based Polish cohort (42.0%), previously published by Kozak-Szkopek et al. [2]. It was shown, that the occurrence of chronic pain increases with age, reaching a frequency of 62% in the group over 75 years old in the United Kingdom population [39]. In line, Docking et al. [40] reported an over 60% occurrence of chronic pain, both in rural and urban subjects who were 55 years old and over, and had been served by general practitioners from Scotland (64.6% and 65.8%, respectively). A lower prevalence was observed by Dahlhmer et al. [41] in residents of the United States of America. In the age group of 65–84-year-old subjects, chronic pain was declared by 27.6%, and 33.6% among the oldest (≥85 years old). These differences may be explained by various definitions and methods used for chronic pain assessments.

The frequency of chronic pain was related to inflammation. In our study, there was a 6.9 percentage points difference (95% CI: 2.0–11.8%) in the occurrence of chronic pain between subjects with significant inflammation in comparison to subjects with mild or no inflammation (48.7% vs. 41.8%; *p* < 0.01). Thus, only significant inflammatory status may be considered as a risk factor for chronic pain in older adults. However, the observed association in this cross-sectional study precludes assessment of the mechanism linking inflammation and chronic pain. Nevertheless, inflammatory status was more related to the occurrence of CWP than CRegP, which supports the existence of inflammation-driven pathways in the pathogenesis of fibromyalgia [42]. It should be stressed that fibromyalgia does not induce inflammation itself. On the other hand, the high prevalence of osteoarthritis in the elderly population continues to be considered as one of the most significant sources of pain. Contrary to the previous paradigm, which linked osteoarthritis solely to mechanical changes, the inflammatory response is now considered as one of the most important cofactors modulating pain and progression of the disease. This recognition may explain the direct link between pain and inflammation demonstrated in our study [43]. In our analysis, the association between CWP and inflammation lost significance in multivariable analysis, probably due to the smaller sample size in comparison to CRegP.

Of note, our study revealed that in addition to the inflammatory status, the female gender, major depression, malnutrition, CAD, COPD, and obesity explain the occurrence of chronic pain.

Referring to the literature, the female gender is usually associated with a more frequent occurrence of chronic pain [44,45]. Our analysis also supports previous findings concerning the greater prevalence of chronic pain in subjects with depression [38,46]. The association between chronic pain and depression is certainly bidirectional. Chronic pain may participate in the development of depression, while depression increases the reported frequency of chronic pain [44]. Additionally, the literature suggests that central hyperexcitability in depressive disorders, frequently related to neuroinflammation, participates in the perpetuation of chronic pain. In Larsson’s [45] paper analyzing chronic pain in older Swedish people (aged ≥ 65 years), 38.8% of people with chronic pain also struggled with depression. Our multivariable models revealed that major depression symptoms are more related to CWP. These data emphasize the need for screening for depression in older people with chronic pain, as its therapy without antidepressants may not be effective in this subset of patients.

Malnutrition is strongly associated with inflammation. Stumpf et al. [47] determined inflammation as the main cause of poor appetite and the stimulation of numerous catabolic processes, leading to malnutrition. The mutual relationship between inflammation and malnutrition ultimately contributes to the development and perpetuation of chronic pain [48]. According to our data, malnutrition is more associated with CWP than CRegP.

It is much more difficult to explain why CAD is associated with both CWP and CRegP. As the disease is caused by atherosclerosis, its occurrence is associated with systemic inflammation and other predisposing clinical conditions, including obesity, and its complications, and smoking. There is much experimental evidence linking inflammation with the expression of osteogenic factors and vascular-smooth-muscle cells transition into osteoblast-like cells, instability, and the calcification of atherosclerotic plaques [49]. Moreover, CAD increases the risk of peripheral artery disease, which is manifest by intermittent claudication—a CRegP subtype restricted to the lower legs. Another explanation for our findings is the fact that chronic pain, including both CRegP and CWP, serves as a significant trigger for sympathetic nerve stimulation. This stimulation may, at least partially, contribute to the exacerbation of CAD, hypertension, and CHF. In other words, chronic pain may predispose individuals to the development of cardiovascular events rather than the other way around [50].

Obesity has many negative health implications, including the development of persistent pain [51]. Yoo et al. [52] showed that the number of women suffering from CWP increased alongside body fat depot. Importantly, it has been shown that people with obesity have an aggravated perception of chronic pain in the course of fibromyalgia [53]. The impact of obesity on chronic pain is multifactorial with biomechanical, metabolic, genetic, and social links [51]. Local inflammation in the course of osteoarthritis related to long-lasting joint overload, enhanced by adipose-derived proinflammatory cytokines, plays a huge role in the development of CRegP [54]. In addition, obesity is bidirectionally related to depression [55]. In our study, the association between obesity, CRegP, and occupational physical activity in the past, but not CWP, supports the hypothesis linking obesity and osteoarthritis-related pain.

The diseases frequently co-occurring with chronic pain, besides depression, are chronic pulmonary diseases, mostly COPD [56], which is strongly related to smoking. Most likely, this association is caused by the prevailing systemic inflammation [57]. In our study, we showed a correlation between chronic pulmonary diseases and CWP, consistent with previous reports.

The main limitations of our study are restricted data on chronic pain characteristics, resulting from the study methodology, and lack of information about the chronic pain causes, especially inflammatory joint diseases. Surprisingly, the analysis of medication showed low utilization of GCS and DMARDs in participants with rheumatoid diseases. In addition, cross-sectional assessment of inflammatory markers (CRP and IL-6) may result from transient subclinical infections, that may somehow disturb our analysis.

## 5. Conclusions

1. Significant inflammation is associated with increased occurrence of CWP in older adults. This supports the existence of an inflammatory-driven pathway of pain-sensing in subjects suffering from this type of chronic pain.
2. Female sex and major depression are the most significant associates of CWP, while malnutrition is the most significant associate of CRegP. In line with our findings, older adults with CWP should be screened for depression, while those with CRegP should be screened for malnutrition.

## Figures and Tables

**Table 1:** Characteristics of the study group [N = 3473] stratified for the occurrence and severity of inflammation and chronic pain.

Inflammation:	Without Inflammation			With Mild Inflammation			With Significant Inflammation			ANOVA or the Log-Linear Analysis		
		Chronic Pain			Chronic Pain			Chronic Pain		p_A_	p_B_	p_C_
	All	YES	NO	All	YES	NO	All	YES	NO			
N [%]	2016 (58.1)	828 (41.1)	1188 (59.7)	1001 (28.8)	433 (43.3)	568 (56.7)	456 (13.1)	222 (48.7)	234 (51.3)			
Women [N; %]	942 (46.7)	447 (54.0) ^#^	495 (41.7)	501 (50.1)	250 (57.7) ^#^	251 (44.2)	206 (45.2)	121 (54.5) ^#^	85 (36.3)	0.13	<0.001	0.52
Age [years]	78 ± 8	78 ± 8	78 ± 8	78 ± 9	78 ± 8	78 ± 9	79 ± 8	79 ± 8	80 ± 9	<0.001	0.30	0.77
65–74 years [N; %]	813 (40.3)	336 (40.6)	477 (40.1)	385 (38.4)	166 (38.3)	219 (38.6)	147 (32.2)	73 (32.9)	74 (31.6)	<0.001	0.58	0.51
75–84 years [N; %]	695 (34.5)	282 (34.0)	413 (34.8)	332 (33.2)	156 (36.0)	176 (31.0)	152 (33.3)	77 (34.7)	75 (32.0)			
≥85 years [N; %]	508 (25.2)	210 (25.4)	298 (25.1)	284 (28.4)	111 (25.7)	173 (30.4)	157 (34.5)	72 (32.4)	85 (36.4)			
Rural area residence [N; %]	1257 (62.4)	511 (61.7)	746 (62.8)	567 (56.6)	240 (55.4)	327 (57.6)	257 (56.4)	121 (54.5)	136 (58.1)	<0.01	0.24	0.88
Blue-collar [N; %]	1001 (54.6)	418 (55.0)	583 (54.4)	512 (55.8)	226 (57.5)	286 (54.5)	265 (61.8)	127 (61.0)	138 (62.4)	<0.001	<0.05	0.72
White-collar [N; %]	596 (32.5)	236 (31.1)	360 (33.6)	249 (27.1)	91 (23.2) *	158 (30.1)	100 (23.3)	48 (23.1)	52 (23.5)			
Farmer [N;%]	235 (12.8)	106 (13.9)	129 (12.0)	157 (17.1)	76 (19.3)	81 (15.4)	64 (14.9)	33 (15.9)	31 (14.1)			
Occupational physical activity in the past [N; %]	1145 (61.8)	501 (65.7) ^$^	644 (59.1)	563 (61.0)	254 (65.1) *	309 (58.0)	305 (70.9)	148 (71.5)	157 (70.4)	<0.001	<0.001	0.57
Personal income												
Low [N; %]	875 (48.9)	357 (48.1)	518 (49.4)	424 (48.4)	177 (46.1)	247 (50.1)	216 (54.1)	105 (54.1)	111 (54.2)	<0.001	<0.01	0.94
Moderate [N; %]	695 (38.8)	311 (41.9 ) *	384 (36.6)	387 (44.1)	181 (47.1)	206 (41.8)	148 (37.1)	76 (39.2)	72 (35.1)			
High [N; %]	200 (12.3)	74 (10.0) *	146 (14.0)	66 (7.5)	26 (6.8)	40 (8.1)	35 (8.8)	13 (6.7)	22 (10.7)			
Active smokers [N; %]	163 (8.1)	62 (7.5)	101 (8.5)	110 (11.0)	46 (10.6)	64 (11.3)	63 (13.8)	29 (13.1)	34 (14.5)	<0.01	<0.01	0.82
Past smokers [N; %]	730 (36.2)	283 (34.2)	447 (37.6)	350 (35.0)	137 (31.6)	213 (37.5)	153 (33.6)	65 (29.3)	88 (37.6)			
BMI [kg/m^2^]	27.8 ± 4.5	28.3 ± 4.8 ^#^	27.4 ± 4.3	29.3 ± 5.3	29.9 ± 5.6 ^$^	28.9 ± 5.0	28.5 ± 5.7	28.8 ± 5.8	28.1 ± 5.5	<0.001	<0.001	0.89
Overweight [N; %]	871 (44.0)	341 (45.0)	530 (45.3)	363 (37.4)	149 (35.3)	214 (40.0)	169 (39.5)	81 (39.1)	88 (39.8)	<0.001	<0.001	0.95
Obesity [N; %]	557 (28.1)	263 (32.4) ^#^	294 (25.1)	410 (42.2)	193 (45.7)	217 (39.5)	146 (34.1)	76 (36.7)	70 (31.7)			
Visceral obesity [N; %]	1617 (81.0)	679 (82.7)	938 (79.8)	843 (85.4)	366 (85.5)	477 (85.3)	360 (80.9)	181 (84.2)	179 (77.8)	<0.01	<0.05	<0.05
Risk of malnutrition [N; %]	774 (39.7)	326 (40.7)	448 (38.9)	372 (38.8)	171 (40.7)	201 (37.3)	206 (49.5)	100 (49.0)	106 (50.0)	<0.001	<0.001	0.67
Malnutrition [N; %]	189 (9.7)	105 (13.1) ^#^	84 (7.3)	76 (7.9)	51 (12.1) ^#^	25 (4.6)	55 (13.2)	34 (16.7) *	21 (9.9)			
Vigorous exercise in the past year [N; %]	1514 (75.8)	602 (73.3) *	912 (77.6)	705 (70.8)	279 (64.6) ^#^	426 (75.5)	273 (60.3)	129 (58.6)	144 (61.8)	<0.001	<0.001	0.15
Dependent in IADL [N; %]	34 (17.6)	156 (19.3) ^#^	192 (16.4)	245 (25.0)	132 (31.1) ^#^	113 (20.4)	157 (35.4)	76 (35.8)	81 (35.1)	<0.001	<0.001	<0.05
Certificate of disability [N; %]	560 (28.0)	269 (33.0) ^#^	291 (24.6)	284 (28.6)	141 (33.1) ^$^	143 (25.3)	138 (30.7)	78 (35.6)	60 (26.0)	0.52	<0.001	0.96
Medical rehabilitation in the past 5 years [N; %]	568 (28.7)	286 (35.1) ^#^	282 (24.2)	251 (25.3)	126 (29.3) *	125 (22.3)	99 (22.0)	64 (29.2) ^#^	35 (15.2)	<0.01	<0.001	0.24
Management by specialists in the past 5 years [N; %]	1215 (61.4)	536 (65.9) ^#^	679 (58.2)	597 (60.4)	271 (63.2)	326 (58.3)	257 (57.2)	130 (59.6)	127 (55.0)	0.27	<0.001	0.66
Hospitalization in the past 5 years [N; %]	1037 (53.5)	442 (55.5)	595 (52.1)	507 (52.3)	241 (57.1) ^$^	266 (48.6)	273 (61.6)	136 (63.8)	137 (60.0)	<0.01	<0.01	0.44
Lab tests in the last 3 years [N; %]	1126 (57.6)	499 (61.8) ^$^	627 (54.6)	528 (53.9)	245 (57.7) *	283 (51.0)	242 (54.4)	119 (64.8)	123 (54.0)	0.12	<0.001	0.46
Upper extremity pain [N; %]	270 (32.6)	270 (32.6)	–	142 (32.8)	142 (32.8)	–	78 (35.1)	78 (35.1)	–	0.77	–	–
Lower extremity pain [N; %]	589 (71.1)	589 (71.1)	–	336 (77.6)	336 (77.6)	–	163 (73.4)	163 (73.4)	–	<0.05	–	–
Axial pain [N; %]	534 (64.5)	534 (64.5)	–	261 (60.3)	261 (60.3)	–	133 (59.9)	133 (59.9)	–	0.23	–	–
Chronic widespread pain [N; %]	170 (20.5)	170 (20.5)	–	84 (19.4)	84 (19.4)	–	52 (23.4)	52 (23.4)	–	0.48	–	–
Chronic regional pain [N; %]	658 (79.5)	658 (79.5)	–	349 (80.6)	349 (80.6)	–	170 (76.6)	170 (76.6)	–		–	–
Pain intensity—VAS [pts]	6.2 ± 2.0	–	–	6.4 ± 2.0	–	–	6.3 ± 2.2	–	–	0.28	–	–
VAS ≥ 5 [pts]	650 (79.7)	–	–	356 (83.4)	–	–	172 (78.9)	–	–	0.23	–	–

Statistical significance of subjects with pain vs. those without pain * *p* < 0.05, ^$^ *p* < 0.01, ^#^ *p* < 0.001; p_A_—statistical significance for inflammation (ANOVA) or interaction of inflammation with analyzed parameter (log-linear analysis); p_B_—statistical significance for pain severity or interaction of pain severity with analyzed parameter; p_C_—statistical significance for interaction in ANOVA or interaction of inflammation, pain severity and analyzed parameter; IADL—The Lawton Instrumental Activities of Daily Living Scale; VAS—Visual Analog Scale.

**Table 2:** Comorbidity, laboratory findings, and medication in the study group [N = 3473] stratified for the occurrence and severity of inflammation and chronic pain.

Inflammation:	Without Inflammation			With Mild Inflammation			With Significant Inflammation			ANOVA or the Log-Linear Analysis		
		Chronic Pain			Chronic Pain			Chronic Pain		p_A_	p_B_	p_C_
	All	YES	NO	All	YES	NO	All	YES	NO			
N [%]	2016 (58.1)	828 (41.1)	1188 (59.7)	1001 (28.8)	433 (43.3)	568 (56.7)	456 (13.1)	222 (48.7)	234 (51.3)			
Comorbidity:												
Diabetes [N; %]	441 (21.9)	196 (23.8)	245 (20.7)	241 (24.1)	108 (24.9)	133 (23.5)	100 (22.0)	45 (20.4)	55 (23.5)	0.39	0.21	0.35
Hypertension [N; %]	1464 (72.7)	613 (74.0)	851 (71.7)	773 (77.4)	341 (79.1)	432 (76.2)	336 (73.8)	161 (72.8)	175 (74.8)	<0.05	0.19	0.57
Coronary artery disease [N; %]	443 (22.0)	211 (25.5) ^$^	232 (19.5)	213 (21.3)	107 (24.7) *	106 (18.9)	90 (19.7)	47 (21.2)	43 (18.4)	0.59	<0.001	0.79
Congestive heart failure [N; %]	77 (3.9)	30 (3.7)	47 (4.0)	52 (5.3)	21 (4.9)	31 (5.5)	57 (12.9)	20 (9.5) *	37 (16.0)	<0.001	0.24	0.35
Chronic pulmonary disease [N; %]	322 (16.0)	153 (18.6) ^$^	169 (14.3)	190 (19.0)	100 (23.1) ^$^	90 (18.8)	108 (23.9)	53 (24.1)	55 (23.7)	<0.001	<0.001	0.26
Hypercholesterolemia [N; %]	1550 (76.9)	641 (77.4)	909 (76.5)	763 (76.2)	336 (77.6)	427 (75.2)	283 (62.1)	134 (60.4)	149 (63.7)	<0.001	0.85	0.53
Hypertriglyceridemia [N; %]	498 (24.7)	211 (25.5)	287 (24.2)	301 (30.1)	148 (34.2) *	153 (26.9)	99 (21.7)	50 (22.5)	49 (20.9)	<0.001	<0.05	0.28
Hyperuricemia [N; %]	418 (21.0)	168 (20.5)	250 (21.3)	325 (33.0)	148 (34.7)	177 (31.7)	161 (36.0)	81 (37.5)	80 (34.6)	<0.001	0.37	0.53
History of cancer [N; %]	94 (4.7)	36 (4.4)	58 (4.9)	45 (4.5)	24 (5.5)	21 (3.7)	27 (5.9)	17 (7.7)	10 (4.3)	0.42	0.31	0.16
Any depression	515 (27.6)	246 (32.3) ^#^	269 (24.4)	299 (31.8)	160 (38.7) ^#^	139 (26.4)	154 (39.0)	91 (45.7) ^$^	63 (32.1)	<0.001	<0.001	1.00
Major depression	84 (4.5)	46 (6.0) ^$^	38 (3.4)	63 (6.7)	37 (9.0) *	26 (4.9)	32 (8.1)	20 (10.1)	12 (6.1)	<0.001	<0.001	1.00
Anti-CCP positive [N/assessed; %]	14/748 (1.9)	4/305 (1.3)	10/443 (2.3)	15/378 (4.0)	7/154 (4.6)	8/224 (3.6)	9/159 (5.7)	5/73 (6.8)	4/86 (4.7)	<0.05	0.87	0.51
Hb [g/dL]	13.8 ± 1.4	13.8 ± 1.4	13.8 ± 1.5	13.7 ± 1.6	13.7 ± 1.6	13.6 ± 1.6	13.9 ± 1.4	13.9 ± 1.5	13.8 ± 1.3	0.13	0.19	0.78
Hb < 11.0 g/dL [N; %]	52 (3.1)	21 (3.1)	31 (3.1)	33 (4.0)	11 (3.1)	22 (4.7)	7 (1.8)	4 (2.1)	3 (1.5)	0.13	0.58	0.57
Albumin [g/L]	43.4 ± 3.0	43.4 ± 3.0	43.4 ± 3.0	42.6 ± 3.1	42.7 ± 3.2	42.6 ± 3.0	40.9 ± 3.7	40.6 ± 4.0	41.1 ± 3.5	<0.001	0.21	0.26
Albumin < 35 g/L [N; %]	18 (0.9)	11 (1.3)	7 (0.6)	13 (1.3)	8 (1.8)	5 (0.9)	30 (6.6)	16 (7.2)	14 (6.0)	<0.001	<0.05	0.54
eGFR [mL/min/L.73 m^2^]	67.4 ± 16.9	67.8 ± 17.0	67.0 ± 16.9	65.2 ± 18.7	65.0 ± 19.6	65.4 ± 17.9	62.5 ± 20.0	61.7 ± 21.3	63.4 ± 18.6	<0.001	0.55	0.36
<45 mL/min/L.73 m^2^ [N; %]	184 (9.1)	79 (9.6)	105 (8.8)	131 (31.1)	69 (15.9) *	62 (10.9)	80 (17.5)	44 (19.8)	36 (15.4)	<0.001	<0.05	0.35
ACR ≥ 30 mg/g [N; %]	252 (13.0)	100 (12.6)	152 (13.3)	163 (17.0)	80 (19.1)	83 (15.4)	109 (25.3)	54 (26.2)	55 (24.6)	<0.001	0.34	0.33
Hematuria [N; %]	142 (7.9)	59 (7.9)	83 (7.8)	83 (9.7)	36 (9.5)	47 (9.8)	67 (17.0)	35 (18.7)	32 (15.5)	<0.001	0.52	0.74
CRP [mg/dL]	1.3 (0.8–2.0)	1.3 (0.8–2.0)	1.3 (0.8–2.0)	4.6 (3.7–6.4)	4.7 (3.7–6.4)	4.6 (3.6–6.3)	15.0 (10.9–24.1)	14.8 (11.1–22.8)	15.2 (10.7–24.2)	<0.001	0.75	0.89
IL-6 [pg/mL]	1.8 (1.2–2.7)	1.8 (1.2–2.7)	1.8 (1.3–2.7)	2.8 (1.8–4.1)	2.8 (1.9–4.0)	2.7 (1.8–4.2)	7.4 (4.1–11.0))	7.7 (4.3–11.0)	7.1 (3.8–10.5)	<0.001	0.27	0.89
Uric acid [mg/dL]	5.2 (4.4–6.1)	5.1 (4.3–6.1)	5.2 (4.4–6.1)	5.6 (4.6–6.7)	5.5 (4.6–6.6)	5.6 (4.7–6.7)	5.8 (4.7–6.8)	5.7 (4.7–6.8)	5.8 (4.6–6.8)	<0.001	0.07	0.44
Hyperuricemia	418 (21.0)	168 (20.5)	250 (21.3)	325 (33.0))	148 (34.7)	177 (31.7)	161 (36.0)	81 (37.5)	80 (34.6)	<0.05	0.37	1.00
Medication:												
Aspirin [N; %]	679 (34.0)	276 (33.7)	403 (34.3)	327 (33.2)	146 (34.2)	181 (32.4)	149 (33.33)	71 (32.9)	78 (33.8)	0.89	0.98	0.79
NSAIDs [N; %]	282 (14.1)	194 (23.7) ^#^	88 (7.5)	152 (15.4)	104 (24.4) ^#^	48 (8.6)	89 (19.9)	68 (31.5) ^#^	21 (9.1)	<0.01	<0.001	0.69
Glucocorticoids [N; %]	16 (0.8)	10 (1.2)	6 (0.5)	7 (0.7)	3 (0.7)	4 (0.7)	10 (2.2)	7 (3.2)	3 (1.3)	<0.05	<0.05	0.59
DMARDs [N; %]	4 (0.2)	0	4 (0.3)	2 (0.4)	2 (0.5)	2 (0.4)	2 (0.4)	1 (0.5)	1 (0.4)	0.33	0.55	0.40

Statistical significance pain vs. no pain * *p* < 0.05, ^$^ *p* < 0.01, ^#^ *p* < 0.001; p_A_—statistical significance for inflammation (ANOVA) or interaction of inflammation with analyzed parameter (log-linear analysis); p_B_—statistical significance for pain severity or interaction of pain severity with analyzed parameter; p_C_—statistical significance for interaction in ANOVA or interaction of inflammation, pain severity and analyzed parameter; ACR—albumin/creatinine ratio; DMARDs—disease-modifying anti-rheumatic drugs; eGFR—estimated glomerular filtration rate.

**Table 3:** The analysis of factors associated with the occurrence of chronic pain as well as widespread and regional pain. Results of univariable logistic regression analysis including inflammatory status.

Inflammation:	Chronic Pain [N = 1483]			Chronic Widespread Pain [N = 306]			Chronic Regional Pain [N = 1177]		
	OR	±95% CI	*p*	OR	±95% CI	*p*	OR	±95% CI	*p*
Women	1.71	1.50–1.96	<0.001	3.50	2.69–4.56	<0.001	1.44	1.25–1.67	<0.001
65–74 years	Ref.			Ref.			Ref.		
75–84 years	1.04	0.89–1.22	0.64	0.93	0.71–1.24	0.64	1.07	0.90–1.27	0.43
≥85 years	0.95	0.80–1.12	0.52	0.81	0.60–1.10	0.18	0.99	0.82–1.18	0.89
Rural area residence	0.92	0.80–1.06	0.24	0.98	0.76–1.25	0.86	0.91	0.79–1.06	0.22
Occupational physical activity in the past	1.31	1.13–1.51	<0.001	1.38	1.05–1.80	<0.05	1.30	1.11–1.52	<0.01
Personal income									
Low	Ref.			Ref.			Ref.		
Average	0.85	0.73–0.99	<0.05	0.53	0.40–0.69	<0.001	0.97	0.82–1.14	0.71
High	0.63	0.49–0.82	<0.001	0.41	0.25–0.68	<0.001	0.71	0.54–0.93	<0.05
Active smokers	0.92	0.73–1.15	0.45	0.69	0.44–1.10	0.12	0.97	0.76–1.24	0.82
Obesity	1.37	1.19–1.58	<0.001	1.31	1.01- 1.69	<0.05	1.38	1.19–1.62	<0.001
Malnutrition	2.10	1.66–2.65	<0.001	2.61	1.83–3.73	<0.001	1.96	1.53–2.52	<0.001
Comorbidity:									
Diabetes	1.11	0.94–1.30	0.21	1.35	1.03–1.78	<0.05	1.04	0.88–1.24	0.63
Hypertension	1.11	0.95–1.29	0.18	1.29	0.97–1.72	0.08	1.06	0.90–1.25	0.46
Coronary artery disease	1.38	1.17–1.62	<0.001	1.47	1.11–1.94	<0.01	1.36	1.14–1.61	<0.01
Congestive heart failure	0.83	0.61–1.12	0.22	0.84	0.49–1.48	0.58	0.82	0.59–1.14	0.23
Chronic pulmonary disease	1.39	1.17–1.66	<0.001	1.81	1.36–2.41	<0.001	1.28	1.06–1.55	<0.01
Hypercholesterolemia	1.02	0.87–1.18	0.84	1.10	0.83–1.46	0.52	0.99	0.84–1.16	0.88
Hypertriglyceridemia	1.17	1.003–1.3	<0.05	1.24	0.95–1.62	0.12	1.15	0.68–1.36	0.09
Hyperuricemia	1.07	0.92–1.25	0.37	1.08	0.82–1.42	0.56	1.07	0.90–1.26	0.43
History of cancer	1.17	0.86–1.60	0.33	1.33	0.79–2.24	0.28	1.12	0.80–1.57	0.50
Any depression	1.63	1.40–1.90	<0.001	2.42	1.87–3.11	<0.001	1.46	1.24–1.72	<0.001
Major depression	2.09	1.54–2.85	<0.001	3.74	2.40–5.80	<0.001	1.73	1.23–2.42	<0.01
Anti-CCP positive	1.03	0.53–1.98	0.93	0.93	0.27–3.15	0.91	1.05	0.52–2.11	0.88
Hb < 11.0 g/dL	0.88	0.57–1.34	0.55	0.92	0.43–1.96	0.84	0.86	0.54–1.37	0.54
Albumin < 35 g/L	1.82	1.09–3.04	<0.05	1.76	0.76–4.10	0.19	1.83	1.07–3.14	<0.05
eGFR < 45 mL/min/L.73 m^2^	1.31	1.06–1.61	<0.05	1.02	0.69–1.52	0.91	1.38	1.11–1. 72	<0.01
ACR ≥ 30 mg/g	1.09	0.91–1.32	0.34	0.83	0.57–1.19	0.31	1.17	0.96–1.43	0.11
Hematuria	1.08	0.85–1.38	0.54	1.08	0.70–1.69	0.72	1.09	0.84–1.42	0.50
Hyperuricemia	1.07	0.92–1.25	0.37	1.08	0.83–1.42	0.56	1.07	0.91–1.26	0.43
Medication:	1,07								
Vigorous exercise in the past years	0.72	0.62–0.84	<0.001	0.68	0.52–0.88	<0.01	0.73	0.62–0.86	<0.001
Without inflammation	Ref.			Ref.			Ref.		
With mild inflammation	1.09	0.94–1.27	0.25	1.04	0.78–1.37	0.80	1.11	0.94–1.31	0.20
With significant inflammation	1.36	1.11–1.67	<0.01	1.55	1.10–2.018	<0.05	1.31	1.05–1.63	<0.05

ACR—albumin/creatinine ratio; eGFR—estimated glomerular filtration rate; CI—confidence interval.

**Table 4:** Factors explaining the occurrence of pain and inflammation in the study population. The results of multivariable logistic regression analyses.

	Chronic Pain N = 1483			Chronic Widespread Pain N = 306			Chronic Regional Pain N = 1177		
	OR	±95% CI	*p*	OR	±95% CI	*p*	OR	±95% CI	*p*
Women	1.72	1.47–2.01	<0.001	3.96	2.94–5.34	<0.001	1.41	1.20–1.66	<0.001
Obesity	1.28	1.08–1.51	<0.01	–			1.36	1.14–1.61	<0.001
Malnutrition	1.62	1.22–2.15	<0.01	1.75	1.14–2.70	<0.05	2.00	1.52–2.62	<0.001
Occupational physical activity in the past	1.33	1.13–1.56	<0.001	1.50	1.12–2.02	<0.01	1.33	1.13–1.57	<0.01
Coronary artery disease	1.41	1.17–1.70	<0.001	1.56	1.13–2.17	<0.01	1.36	1.13–1.65	<0.01
Chronic pulmonary disease	1.32	1.08–1.61	<0.01	1.91	1.12–2.02	<0.01	–		
Major depression	1.67	1.17–2.38	<0.01	2.85	1.68–4.82	<0.001	–		
Inflammation									
no	Ref.			Ref.			Ref.		
mild	1.03	0.86–1.22	0.77	–			1.07	0.90–1.28	0.43
significant	1.28	1.01–1.62	<0.05	–			1.27	1.002–1.62	<0.05
GOF			0.48	–		0.76			0.88
VIF; mean (range)	1.43	1.18–1.79		1.30	1.15–1.53		1.37	1.14–1.70	

GOF—The goodness of Fit; VIF—A variance inflation factor; CI—confidence interval; – means the variable was insignificant and excluded from the final model.

## Data Availability

The data presented in this study are available on request from the corresponding author.

## Supplementary Materials

The following supporting information can be downloaded at: https://www.mdpi.com/article/10.3390/jcm13195870/s1, Table S1: Dependence, disability, and utilization of medical services in relation to pain and inflammation. Results of univariable analyses adjusted for sex—presenting numerical data for the Figure 2.
