# Supplementary material for: Inflammation in Older Poles with Localized and Widespread Chronic Pain—Results from a Population-Based PolSenior Study
Source: J Clin Med. 2024 Oct 1;13(19):5870. doi: 10.3390/jcm13195870 (PMC11478211; doi:10.3390/jcm13195870)
Supplement: Supplementary file 1 [file jcm-13-05870-s001.zip › jcm-3199187-supplementary.pdf]

Supplementary Materials

Table S1. Dependence, disability, and utilization of medical services in relation to pain and inflammation. Results of univariable analyses adjusted for sex.

	Chronic Pain			Inflammation (significant vs mild + no)		
	**OR**	**± 95% CI**	**p**	**OR**	**± 95% CI**	**p**
Dependent in IADL	1.37	1.24–1.52	< 0.001	2.19	1.73–2.77	< 0.001
Certificate of disability	1.51	1.49–1.52	< 0.001	1.12	1.05–1.20	< 0.01
Medical rehabilitation in the past 5 yrs	1.65	1.48–1.84	< 0.001	0.74	0.64–0.86	< 0.001
Management by specialists in the past 5 yrs	1.31	1.26–1.36	< 0.001	0.85	0.70–1.04	0.12
Hospitalization in the past 5 yrs	1.23	1.11–1.37	< 0.001	1.42	1.30–1.55	< 0.001
Lab tests performed in the last 3 yrs	1.28	1.21–1.36	< 0.001	0.92	0.74–1.14	0.47
Major depression	1.87	1.12–3.12	< 0.05	1.59	1.50–1.69	< 0.001
Medication:						
NSAIDs	3.84	3.69–4.00	< 0.001	1.46	1.16–1.84	< 0.01
Glucocorticoids	2.08	1.52–2.85	< 0.001	2.94	1.21–7.14	< 0.05

DMARDS were not included in the analysis due to the low number of prescribed subjects (N=10); lADL = The Lawton Instrumental Activities of Daily Living Scale; NSAIDs = non-steroidal anti-inflammatory drugs
